# Prediction error in implicit adaptation during visually- and memory-guided reaching tasks

**DOI:** 10.1038/s41598-024-59169-2

**Published:** 2024-04-13

**Authors:** Kosuke Numasawa, Takeshi Miyamoto, Tomohiro Kizuka, Seiji Ono

**Affiliations:** 1 Graduate School of Comprehensive Human Sciences, University of Tsukuba, 1-1-1, Tennodai, Tsukuba, Ibaraki 305-8574 Japan; 2 Graduate School of Informatics, Nagoya University, Furo-cho, Chikusa-ku, Nagoya, 464-8601 Japan; 3 Institute of Health and Sport Sciences, University of Tsukuba, 1-1-1, Tennodai, Tsukuba, Ibaraki 305-8574 Japan

**Keywords:** Visuomotor adaptation, Implicit adaptation, prediction error, EEG, Time–frequency analysis, Human behaviour, Sensorimotor processing

## Abstract

Human movements are adjusted by motor adaptation in order to maintain their accuracy. There are two systems in motor adaptation, referred to as explicit or implicit adaptation. It has been suggested that the implicit adaptation is based on the prediction error and has been used in a number of motor adaptation studies. This study aimed to examine the effect of visual memory on prediction error in implicit visuomotor adaptation by comparing visually- and memory-guided reaching tasks. The visually-guided task is thought to be implicit learning based on prediction error, whereas the memory-guided task requires more cognitive processes. We observed the adaptation to visuomotor rotation feedback that is gradually rotated. We found that the adaptation and retention rates were higher in the visually-guided task than in the memory-guided task. Furthermore, the delta-band power obtained by electroencephalography (EEG) in the visually-guided task was increased immediately following the visual feedback, which indicates that the prediction error was larger in the visually-guided task. Our results show that the visuomotor adaptation is enhanced in the visually-guided task because the prediction error, which contributes update of the internal model, was more reliable than in the memory-guided task. Therefore, we suggest that the processing of the prediction error is affected by the task-type, which in turn affects the rate of the visuomotor adaptation.

## Introduction

Our body movements are maintained accurately to achieve one’s goals even under continuous changes in the internal and external environment. This accuracy is acquired by motor adaptation, which is the process of correcting the motor command based on an error resulting from a previous movement. It is well documented that motor adaptation involves explicit strategy and implicit adaptation^1–4^. The explicit strategy corrects our movements with intent, whereas the implicit adaptation gradually corrects the motor command by error signals and it is presumed to be supported by neuronal plasticity in the cerebellum^4,5^. This motor adaptation is crucial for the recalibration of motor commands because it proceeds slowly and its memory is retained for a long term^6^. It is also known that the implicit adaptation is driven by prediction error, which is the difference between the sensory consequence predicted from an efference copy and the actual sensory feedback^7–9^. Thus, the adaptation appears to be notably affected by the process of prediction error. Most previous studies on motor adaptation of reaching movements have used a visually-guided task. This task is referred to as a reactive response in which the subject is instructed to reach for a target presented in the periphery as quickly as possible. Recently, a memory-guided task has been used to examine more voluntary movements in saccade adaptation. This task requires more cognitive processes compared with the visually-guided task because subjects are required to memorize a visuospatial location of the visual cue and to move toward the remembered target. Thus, the memory-guided task may have prediction errors differently compared to the visually-guided task. In saccade adaptation, which is a typical paradigm to clarify adaptive behavior, saccades adapted by the memory-guided task do not affect the visually-guided saccade^10^. Hence, these neuronal mechanisms are thought to be different between tasks. We hypothesized that the task affects the process of prediction error, which in turn influences implicit adaptation. However, it is still uncertain whether the prediction error induced by the memory-guided task influences motor adaptation, since prediction errors in the memory-guided task can be influenced by cognitive and voluntary processes. Therefore, we tested whether cognitive processes influence prediction errors in the implicit adaptation by comparing the visually- and memory-guided tasks.

Adaptation to a visuomotor rotation, in which the visual feedback of the limb rotates relative to the actual limb position, has been used as a perturbation for studying sensorimotor learning. Since this method induces sensory prediction error, subjects need to modify the feedforward motor command with respect to the direction of rotation. In particular, some studies have suggested that the implicit effect on the adaptation is enhanced by the gradual rotation^11,12^. In the current study, we investigated whether the visually-guided and memory-guided tasks influence prediction error during implicit adaptation using the gradual visuomotor rotation. Furthermore, we evaluated electroencephalography (EEG) during adaptation in order to clarify neural mechanisms specific to each task. An increasing number of evidence with EEG signals has revealed the mechanism of sensorimotor adaptation^13^. Beta-band activity at pre-movement over the motor area is considered to be associated with the implicit adaptation^14,15^, whereas over the frontal area relates to the strategic or cognitive process^16^. In addition, it is suggested that low theta-band activities over the parietal area are linked with processing the visuomotor prediction error along with the cerebellum^17^. Therefore, we hypothesized that EEG activities in these frequency bands predict the task type if the neural mechanisms involved in the visually- and memory-guided tasks are different, as suggested in saccade studies.

## Materials and methods

### Subjects

Eight right-handed male subjects (aged 21–29 years old) with no history of pathologies to the upper limb participated in this study. All the subjects gave their informed consent to participate in the experiment. This study was conducted in accordance with the Declaration of Helsinki, and all protocols were approved by the Research Ethics Committee at the Faculty of Health and Sport Sciences, University of Tsukuba.

### Apparatus

**Figure 1:**
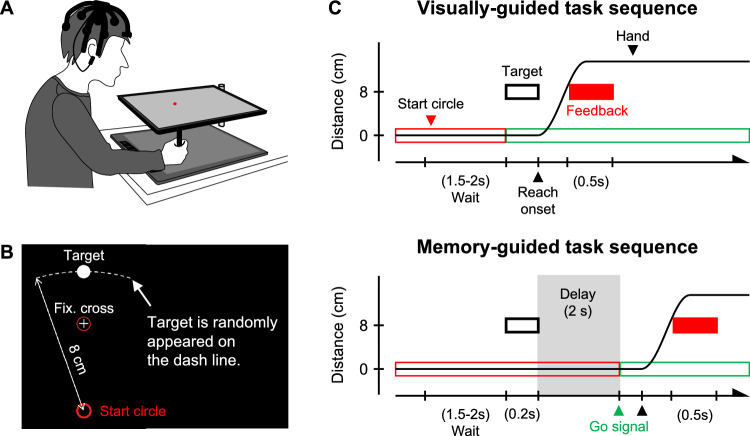
(**A**) Experimental apparatus. Subjects sat in front of a monitor and a pen tablet. They gripped a stylus and controlled a red cursor on the monitor. To evaluate brain activities, they were equipped with electroencephalography (EEG) on their head. (**B**) Arrangement of visual presentation. Visual stimuli consisted of a start circle, a white target, a fixation cross, and the red cursor controlled by subjects. The distance between the target and the start circle was 8 cm, and the target randomly appeared at an angle − 10° to 10° with respect to the centerline, shown as the dashed line in the figure. (**C**) Fundamental task procedures, upper and lower panels show the visually-guided and the memory-guided tasks, respectively. In the visually-guided task, the target appeared 1.5–2 s after subjects move the cursor over the start circle. They were asked to reach for the target as soon as possible when the target was presented. Visual feedback of the actual or modified cursor position was presented when the hand position reached 8 cm from the start circle. In contrast, in the memory-guided task, there was a 2 s delay after the target appeared. Therefore, subjects were required to start reaching movements for a remembered target after a circle around the fixation cross was turned green as soon as possible.

Figure 1A shows the experimental apparatus, which consists of a digitized tablet (Wacom Intuos pro Large, workspace, 311 × 216 mm) to capture the movement of a stylus, and an LCD monitor (HP, X27q QHD; 27 in., 1440 × 2560 pixels, refresh rate 60 Hz) to display visual stimuli and a cursor controlled by the stylus. All stimuli and position data were operated by the Psychophysics Toolbox extensions on MATLAB (The MathWorks Inc., Massachusetts, USA). The x- and y-axes were defined as horizontal and vertical axes, respectively. The subjects sat in front of the monitor placed on a horizontal plane and wore an electrode cap (Quick-20, CGX, San Diego, CA, USA).

### Experimental task

Subjects employed both the visually-guided task and the memory-guided task on separate days. An interval of at least 24 h was set between the two tasks. To avoid the effect of the task order, three subjects started with the visually-guided task, and the remained subjects started with the memory-guided task. Subjects were instructed to manipulate a red cursor on the monitor with the stylus while gazing at a fixation cross throughout one trial. Figure 1C shows the sequences of each task. First, subjects were required to place and stay the cursor over the start circle, then the red circle appeared around the fixation cross. After a random interval between 1.5 and 2 s, a white target appeared at a random position, 8 cm away from the start circle and at an angle of − 10° to 10° with respect to the centerline. In the visually-guided task, subjects were asked to start a reaching movement toward the target as soon and accurately as possible once the circle around the fixation cross turned green with the target appeared. The target disappeared after the onset of the reaching movement. In the memory-guided task, the target was presented for 0.2 s only, and subjects were asked to start a reaching movement when the circle turned green in 2 s after the target disappeared. In both tasks, the cursor feedback was presented when the cursor reached 8 cm from the start circle, and it remained for 0.5 s. Therefore, information on the reaching trajectory was not fed back to subjects, both during and after reaching. In addition, they were instructed to shoot the target without stopping their reaching the target position. If the cursor did not reach 8 cm within 0.3 s, the monitor displayed an instruction “move faster”. These instructions encouraged subjects to move in the target direction as quickly as possible, without considering the distance.

Each of the two tasks consisted of 180 trials, divided into 6 blocks as follows: baseline block (30 trials), adaptation blocks (30 trials × 4), washout block (30 trials), and there were intervals of 1 min 30 s between blocks. The cursor feedback was presented as the actual hand position in the baseline and the washout block. In the adaptation block, the cursor feedback was rotated counterclockwise around the center of the start circle from the actual hand position and its rotation was increased by 0.2° up to 20°. Therefore, the rotation angle reached 20° on the 101st trial and remained at 20° for the following 19 trials. We did not inform subjects that the cursor was rotated. In the washout brock, the cursor rotation was removed, and the visually-guided task was conducted regardless of the task condition. This allowed a comparison of the recovery process from adaptation.

### Behavioral data analysis

The cursor position data were sampled by MATLAB at 60 Hz and filtered offline with a fourth-order Butterworth low-pass filter with a passband of 20 Hz. The hand angle was calculated as the angle error between the target and the actual hand position originating from the center of the start circle at peak y-velocity. We excluded trials in which hand angles deviated more than 3 standard deviations (SD) from a moving mean based on a 5 trial window. Consequently, only two trials were removed from all trials (2880) and the remaining trials were used for the following analysis.

We calculated the SD of hand angles in the baseline block to assess the variability of reaching movements in each task. Reaction time (RT) was defined as the timelapse from the go signal to the first time-point when the y-velocity exceeded 20 mm/s, and movement time (MT) was calculated by a duration of the y-velocity exceeding 20 mm/s. Then, an adaptation rate and a retention rate were estimated by a state-space model applying to hand angles in the adaptation block as following Eq. (1)^11,18–20^;

1 $$e_{t} = r_{t} - { }x_{t}$$

2 $$\begin{array}{*{20}c} {x_{t + 1} = Ax_{t} + Be_{t} } \\ \end{array}$$

where *e*_*t*_ denotes the amplitude of reaching error, *r*_*t*_, indicates the size of cursor rotation, and *x*_*t*_ indicates the internal state of the system on the *t* th trial. The error was defined as the angle between the cursor position and the target position. Equation (2) indicates that internal state *x* changes by a certain proportion of the previous internal state and the experienced error. The retention rate was defined as a coefficient *A*, which means how much of the previous state is retained. Since a coefficient *B* means the amount of change to the error, it was defined as the adaptation rate. These parameters were estimated by the linear regression with the hand angle at *t* + 1 the trial as the dependent variable and the reaching error at *t* th trial as independent variable. We used MATLAB function *regress* to calculate parameters. In addition, the mean absolute error was obtained by the error during the adaptation block and the aftereffect was calculated by the mean hand angle during the washout block.

To examine the effect of the task on the adaptation, we conducted a two-way repeated-measures ANOVA (rmANOVA) for the mean hand angle and the mean absolute error across two task-types (visual, memory) × four blocks (block 1, block 2, block 3, block 4), and for RT and MT across two task-types (visual, memory) × 5 blocks (baseline, block 1, block 2, block 3, block 4). Effect sizes of rmANOVA were reported as partial *η*_*p*_^2^. In addition, we implemented paired *t* tests to the retention rate, the adaptation rate, the SD in the baseline block and the after effect. A significance level was set below *p* < 0.05. The effect size was also calculated as Cohen’s *d* which was defined as small when *d* ≤ 0.2, medium when 0.2 < *d* < 0.8, and large when *d* ≥ 0.8.

### EEG data analysis

The EEG signals were acquired by the 20 channels mounted in electrode caps based on the International Federation 10–20 system (Quick-20, CGX, San Diego, CA, USA). The common reference was located at the left earlobe, and two electrodes were located adjacent to the Fp1 and Fp2 positions as ground. The impedance of each electrode was maintained below 200 kΩ. The impedances with dry electrodes are much higher than those of wet electrodes (usually below 5 kΩ), but this device can obtain the EEG signals well if the impedance is less than 2500 kΩ.

The obtained EEG data were firstly band-pass filtered (0.05–100 Hz) and notch-filtered (50 Hz) offline in Brain Vision Analyzer 2.1 (Brain Products GmbH, Munich, Germany). In the following analysis, these data were processed in MATLAB with the FieldTrip toolbox^21^. Data were segmented into two time periods: − 2 to 2 s of the reaching initiation to evaluate the preparation activity, and − 2 to 2 s of the feedback presentation to assess the visuomotor process. We removed noise components by the independent component analysis (ICA) with the FieldTrip. ICA is a common method to find and subtract artifacts from EEG data^22^. Based on previous EEG studies focusing on motor adaptation^13^, we examined the Fz, C3, and parietal electrodes (P3, Pz, P4). We obtained signal power spectra at a 1 Hz resolution (1–30 Hz) using complex Morlet wavelets on moving 200 ms windows of the filtered signals at 50 ms steps. Then, these power signals were normalized by a decibel conversion relative to the baseline activity (1500–1000 ms before reaching onset for the reaching initiation-related analysis and 100–0 ms before feedback onset for the visual feedback-related analysis, respectively). Finally, these signals were averaged over delta (1–4 Hz), theta (4–8 Hz), alpha (8–13 Hz), and beta (13–30 Hz) frequency bands.

To test whether the two tasks were supported by different EEG activities, we used linear decoders, the support vector machine (SVM), to classify the task type in which the subject engaged based on the power signals. SVM was applied to the power signals 1 s before the reaching initiation and 1 s after the feedback presentation. Then, we tested classification accuracy using leave-one-subject-out cross-validation at each time point. In short, we trained a classifier on seven subjects’ data and then tested on left out subject data. This procedure was repeated with the number of subjects. This cross-validation method is useful for computing classification accuracy with small sample sizes. The statistical significance of decoder performance was determined by a permutation test, where an empirical null distribution of decoder performance was computed by repeating the above classification on data with shuffled relationships between the task type and the power signal (n = 1000)^23,24^. If the decoder performance of the true data exceeded the 95 percentile of the null distribution, the performance was regarded as significantly higher than the chance level (*p* < 0.05). We can obtain the decoder performance considering the multiplicity across the time by calculating the null distribution using the permutation method^25^.

## Results

### Behavioral results

**Figure 2:**
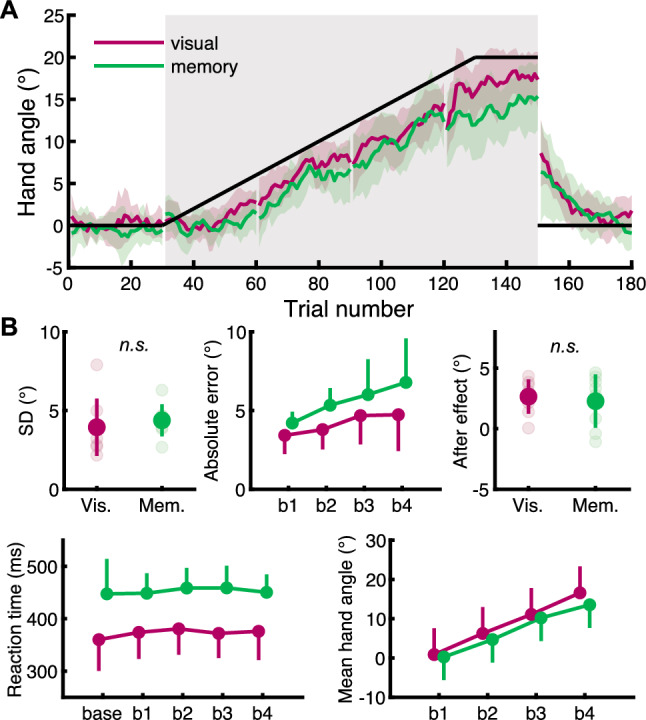
The time course of mean hand angles and the summary of behavioral results. (**A**) Black line shows transitions of the cursor rotation, and the gray shaded area indicates the adaptation block. During adaptation, the cursor rotation increased gradually from the 30th trial to the 130th trial. Red and green traces indicate hand angles calculated by the angle between the target and the actual hand position in the visually-guided and memory-guided tasks, respectively. The traces are presented as moving averages of five trials. (**B**) Red and green circles show mean values, and the colored bars across the circle mean standard deviation. The pale-colored circles indicate each subject’s values. There was a main effect of the task-type on the absolute error, the RT and the mean hand angle. Although a main effect of the block was observed in the absolute error and the mean hand angle, there was a significant interaction only in the mean hand angle.

Figure 2A shows the time course of mean hand angles for the visually-guided task (red) and the memory-guided task (green). Note that these traces are depicted as moving averages of five trials. Figure 2B indicates the summary of behavioral results. There was no difference in the variability of reaching movements between the tasks in the baseline block (*t*_7_ =  − 0.650,* p* = 0.536, *d* = 0.296), indicating that the subjects held the same level of reaching stability regardless of the task type. Additionally, subjects performed similar ballistic movements in each task, since the two-way rmANOVA showed no main effect of the task-type and the block on the MT (task type: *F*_*1,7*_ = 0.556, *p* = 0.480, *η*_*p*_^2^ = 0.074, block: *F*_*4,28*_ = 0.862, *p* = 0.435, *η*_*p*_^2^ = 0.110). In contrast, there was a main effect of the task-type on RT (*F*_*1,7*_ = 17.204, *p* = 0.004, *η*_*p*_^2^ = 0.711), but no effect of the block (*F*_*4,28*_ = 1.238, *p* = 0.318, *η*_*p*_^2^ = 0.150), showing significantly shorter RT in the visually-guided task than in the memory-guided task regardless of adaptive behavior. The result could be involved in the difference in adaptive behavior in the adaptation block. In accordance with this possibility, we found a main effect of the task-type on the mean hand angle (*F*_*1,7*_ = 6.213, *p* = 0.041, *η*_*p*_^2^ = 0.470), and it increased naturally in accordance with the number of blocks (*F*_*3,21*_ = 231.064, *p* < 0.001, *η*_*p*_^2^ = 0.971). There was significant interaction between the task-type and the block (*F*_*3,21*_ = 4.171, *p* = 0.018, *η*_*p*_^2^ = 0.373). These results in the adaptation block are consistent with our hypothesis that the adaptation is affected by the task type. Moreover, a main effect of the task-type and the block on the absolute error was observed (task-type: *F*_*1,7*_ = 8.062, *p* = 0.025, *η*_*p*_^2^ = 0.535, block: *F*_*3,21*_ = 7.266, *p* = 0018, *η*_*p*_^2^ = 0.509), but there was no interaction (*F*_*3,21*_ = 1.124, *p* = 0.335, *η*_*p*_^2^ = 0.138). Since the lower error indicates that subjects reached the target accurately, the result supports the high adaptability in the visually-guided task. Although we expected that the magnitude of aftereffect also depends on the task type if the level of adaptation is modulated by the task, there was no difference between the tasks in the washout block (*t*_7_ = 0.410 *p* = 0.694, *d* = 0.201).

**Figure 3:**
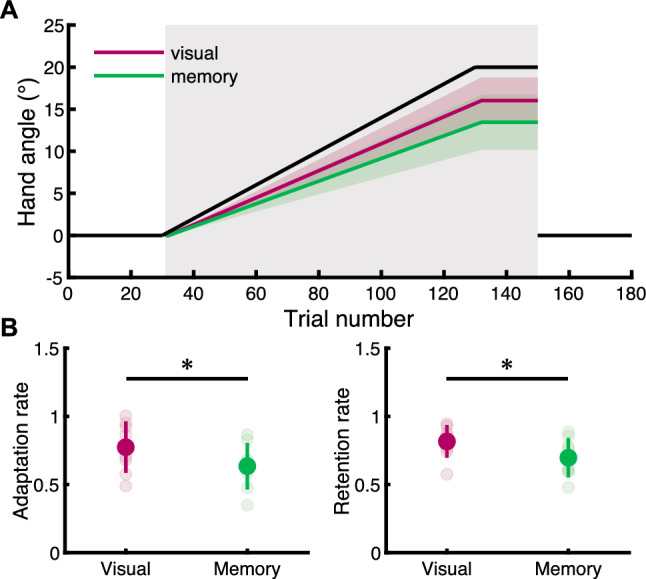
The state-space model in the adaptation block. (**A**) Black line shows transitions of the cursor rotation. Red and green traces indicate the predicted hand angle by the state-space model in each subject. This model fitted hand angles well (visual: *R*^*2*^ = 0.672 ± 0.146, memory: *R*^*2*^ = 0.526 ± 0.141). (**B**) Red and green circles show mean values, and the colored bars across the circle mean standard deviation. The pale-colored circles indicate each subject’s values. Asterisks indicate the statistical significance, as the result of paired *t* test (*p* < 0.05).

Figure 3A shows the predicted hand angles by the state-space model in the adaptation block. This model well fitted the original hand angle for each subject (visual: *R*^*2*^ = 0.672 ± 0.146, memory: *R*^*2*^ = 0.526 ± 0.141). It indicates that hand angles increased in accordance with the cursor rotation, and its slope in the visually-guided task seems to be greater than in the memory-guided task. We found that the adaptation rate and the retention rate calculated from the linear regression in the adaptation block were significantly higher in the visually-guided task than in the memory-guided task (the adaptation rate:* t*_7_ = 2.403, *p* = 0.047, *d* = 0.774, the retention rate: *t*_7_ = 2.791, *p* = 0.027, *d* = 0.895, Fig. 3B). The result suggests that motor memory is well retained in the visually-guided task, in addition to the high adaptation rate.

### Task-type-dependent time–frequency representation

**Figure 4:**
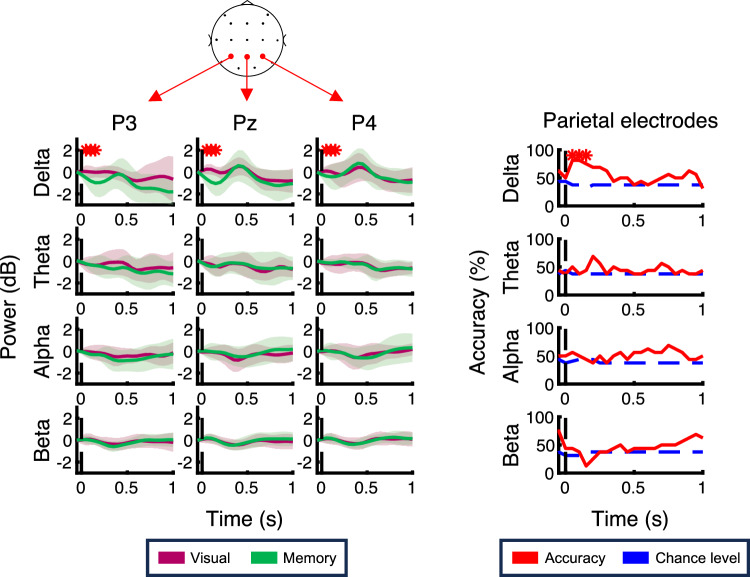
The time course for each frequency-band power and the performance of the task type decoder using the power signals in each band aligned to the feedback onset at parietal electrodes. On the left panel, red and green traces show the time course for powers in the visually-guided and memory-guided tasks, respectively. The colored regions represent the standard deviation. On the right panel, the performance of the task type decoder computed by the support vector machine (SVM) using the power signals at parietal electrodes is presented. The red lines indicate the decoder performance and the blue dashed lines show the chance level, which is the median value of the null distribution generated by the permutation test. Red asterisks mean the decoder performance significantly exceeded the chance level.

**Figure 5:**
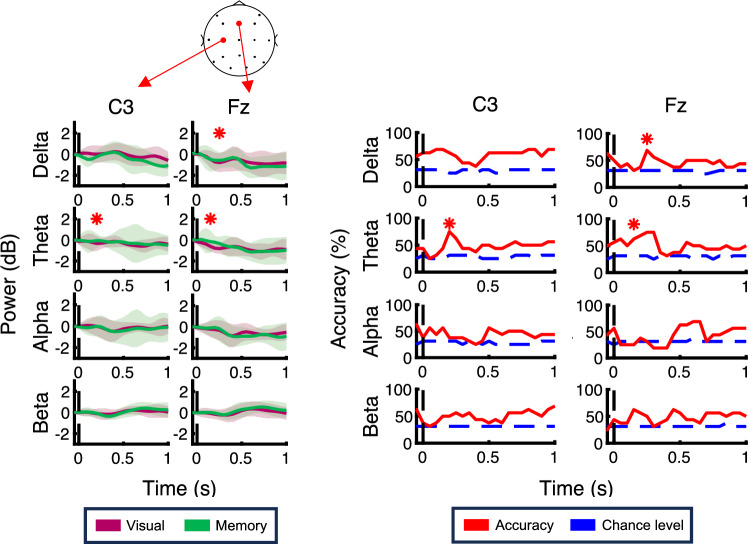
The time course for each frequency-band power and the performance of the task type decoder using the power signals in each band aligned to the feedback onset at the C3 and Fz electrodes are plotted in the same format as Fig. 4.

Figure 4 shows the time course for each frequency-band power and the performance of task type decoder using the power signals in each band aligned to the feedback onset at parietal electrodes. As shown in the figure, in the visually-guided task, the delta-power (1–4 Hz) at all parietal electrodes increased after the feedback, whereas this trend was minor in the memory-guided task. This tendency corresponds to a previous study that the prediction error modulates the low theta-band activity (2–4 Hz)^17^. To confirm this difference depending on the task type, we computed the classification accuracy using the SVM. As a result, the decoder performance exceeded 75% at the time between 50–150 ms, above the statistically computed chance level. In contrast, there was no significance on the performance of the other frequency bands at parietal electrodes. This analysis shows that the task type was encoded as a delta-band activity after the visual feedback at parietal electrodes. Figure 5 shows the results at the C3 and Fz electrodes. Similar activities were observed at the C3 electrode, which is the contralateral side of the reaching arm, but there was no significant decoder performance in the delta-power at the C3. On the other hand, the delta-power at the Fz electrode and the theta-power at Fz and C3 electrodes were immediately decreased in the visually-guided task. These observations were supported by the decoding analysis. The performance exceeded the chance level at 200 ms in the theta-power at the C3 electrode. At the Fz electrode, the significant performance was found at 250 ms in the delta-power and at 150 ms in the theta-power.

**Figure 6:**
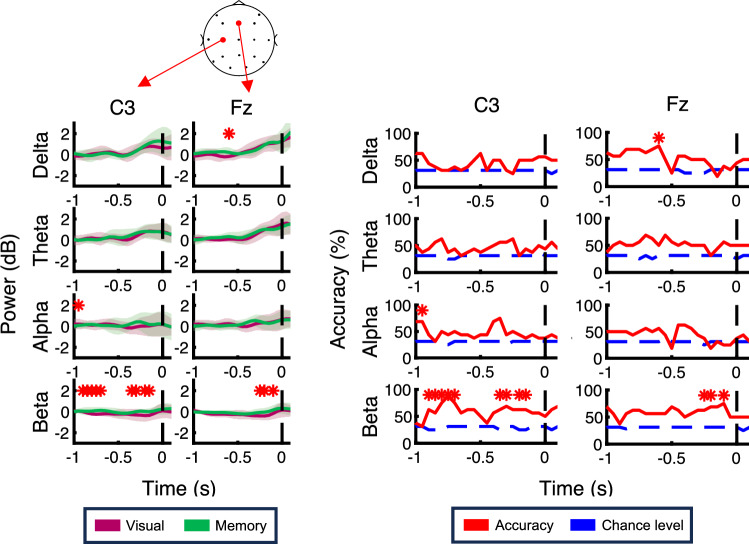
The time course for each frequency-band power and the performance of the task type decoder using the power signals in each band aligned to the reaching onset at the C3 and Fz electrodes are plotted in the same format as Fig. 4.

Next, we focused on activities before reaching onset to evaluate whether the preparation process is affected by the task type. The left panel of Fig. 6 shows the time course for power in each band at the C3 and Fz electrodes. In both electrodes, the beta-power remarkably decreased before reaching movements in the visually-guided task. In fact, the significant performance of the task type decoder at the C3 electrode was found at – 950 ms in the alpha-power and in the − 900 to – 700 ms, − 350 to – 300 ms, and − 200 to − 150 period in the beta-power, as shown in the right panel of Fig. 6. At the Fz electrode, the decoder performance significantly exceeded the chance level at – 600 ms in the delta-power and in the − 250 to − 200 ms and – 100 ms period in the beta-power. The segregation of the two task types in the beta-power appeared to be mainly the result of a decrease in power beginning 900 ms before reaching onset in the visually-guided task.

**Figure 7:**
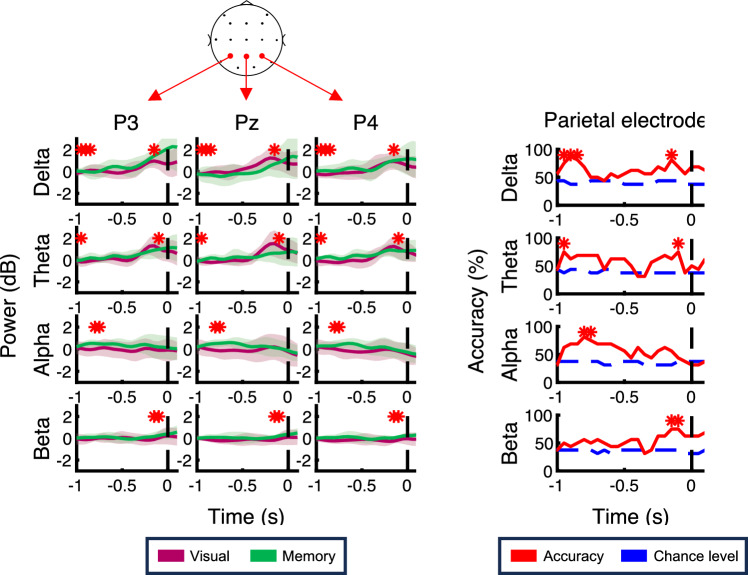
The time course for each frequency-band power and the performance of the task type decoder using the power signals in each band aligned to the reaching onset at parietal electrodes are plotted in the same format as Fig. 4.

Figure 7 shows the time course for each band power and the decoder performance of each band aligned to the reaching onset at parietal electrodes. As with the Fz and C3 electrodes, the beta-power signal in the parietal electrodes appears to be no distinct difference in the broad time range. However, the decoder performance was above the chance level in the − 950 to – 850 ms period in the delta-power, at – 950 ms and – 100 ms in the alpha-power, in the – 800 to – 750 ms period in the alpha-power, and in the – 150 to – 100 ms period in the beta-power. These results indicate that the parietal area is associated with the preparation of adaptive movements as well as the frontal and motor area electrodes.

Taken together, our results showed that the differences in task types inducing motor adaptation are characterized by delta-power signals in the parietal and contralateral central areas immediately after feedback, and by power signals in a broad frequency band, mainly beta-power, in the frontal and contralateral central areas during preparation for reaching.

## Discussion

The purpose of this study was to clarify the difference in the visuomotor adaptation in the visually-guided task and the memory-guided task. We applied the gradual rotation to the visual feedback during the adaptation block and estimated adaptation parameters by the state-space model. Our behavioral results demonstrated that the mean hand angle, adaptation and retention rates were higher in the visually-guided task than in the memory-guided task, and the absolute error during the adaptation block was smaller in the visually-guided task than in the memory-guided task. It is likely that the low adaptation and retention rates in the memory-guided task are caused by higher cognitive processes to reach the target location. Similarly, RT was longer in the memory-guided task than in the visually-guided task. It has been reported that the saccadic eye movements require more time to initiate in the memory-guided task than in the visually-guided task and it involves higher neuronal pathways for memory^26,27^. In the reaching task, RT is similarly extended in the memory-guided task^28^. Therefore, longer RT in the memory-guided task indicates longer processing of visuomotor working memory. It has been reported that the implicit adaptation is based on the sensory prediction error and driven by the cerebellum plasticity change^5,7–9^. Our results demonstrated that the absolute error was smaller in the visually-guided task despite high adaptation and retention rates. This result leads to an assumption that visuomotor adaptation is affected by the certainty of the prediction error. Therefore, the high adaptation and retention rates in the visually-guided could be caused by the reliable prediction error, and it could be associated with the plastic change of the cerebellum. Conversely in the memory-guided task, a higher cognitive process may negatively affect prediction error and cerebellar adaptation. Cognitive effects might be more apparent by testing a larger number of trials after the cursor rotation reaches the maximum value, since the hand angle in the memory-guided does not appear to be saturated. If the angle increases with the number of trials, the memory-guided task may take longer to adapt. Otherwise, the memory-guided task could not properly handle the error.

Our main finding was that two different tasks were classified by the delta band-power (1–4 Hz) associated with the visual feedback at the parietal electrodes. This means that different cortical activities took place depending on the tasks, as the delta power significantly increased in the visually-guided task more than in the memory-guided task. It has been reported that low theta-power (2–4 Hz) is increased by the visuomotor rotation feedback^17^. This previous study suggests low-frequency activity at the parietal area reflects the sensory prediction error because its synchronization carries feedforward influences like the prediction error signal^29^. Our parietal electrodes are located on the posterior parietal cortex (PPC), which is involved in integrating predicted and actual sensory signals^30–32^. Since the predicted sensory signal is thought to be transmitted from the cerebellum to the PPC^33–35^, the PPC activity would be attenuated if the sensory prediction from the cerebellum is consistent with the actual feedback^36,37^. Therefore, we suggest that the PPC is primarily involved in generating the prediction error signal, and the increase in delta power in the PPC in our results could reflect the reliability of sensory prediction error elicited by the visuomotor rotation.

Interestingly, the delta power was larger in the visually-guided task, even though the absolute error was significantly smaller than in the memory-guided task. This discrepancy might be due to the fact that the prediction error does not originate from conscious prediction^17^, and that the adaptation for the visually-guided task is dependent on the implicit adaptation. The reliable prediction error shown in the delta power could lead to the high sensitivity for the sensory perturbation since the sensory prediction is more accurate in the visually-guided task. Indeed, it has been reported that the accuracy of the sensory prediction increases sensitivity to the prediction error^38^. In contrast, the memory-guided task could inhibit cognitive activity associated with prediction. Considering that implicit adaptation is driven by the prediction error, a high adaptation rate in the visually-guided task is consistent with an increase in the delta power, which reflects the reliability of the prediction error. Moreover, a high retention rate could be related to the cerebellum updates because there is a strong connection between the PPC and the cerebellum. Since the gradual rotation task is considered to increase implicit effects^11,12^, our results suggest that a memory-guided task decreases the implicit adaptation. Therefore, the processes of the prediction error in the PPC and plastic changes in the cerebellum that contribute to the implicit adaptation could be affected by the higher-order cognitive processes.

In the phase before the reaching initiation, our results demonstrated that the beta band activities were desynchronized in the visually-guided task at the C3 and the Fz electrodes. The reduction of alpha and beta oscillations is considered to be related to motor preparation^39–41^. It has been reported that the beta reduction in the motor and sensorimotor regions is associated with the level of accurate motor planning in the direction of movements^42,43^. Hence, the current result could be regarded as the reaching planning is more accurate in the visually-guided task than the memory-guided task. Considering that the reliable prediction error originates from accurate motor commands^38^, it is most likely that the high adaptation rate in the visually-guided task is induced by robust sensory prediction based on accurate motor planning. This idea is consistent with the reliable prediction error shown by the increase in delta power in the visually-guided task. Alternatively, the reduction in beta power at the C3 electrode could reflect an update of the motor command. It has been reported that pre-movement beta power decreases in accordance with the size of the error in the sensorimotor adaptation^15,44^. The reduction is particularly large in the early phase of the adaptation trials^14^. Moreover, the beta reduction in the motor area is considered to be due to the integration of sensory information to update motor commands^13,45^. Therefore, the reduction in beta power at the C3 in the visually-guided task could implicate the modulation of the motor planning to adapt the cursor rotation. Furthermore, different visual information could affect the power signals. While subjects were not able to use information related to the target direction around – 1 s before the reaching initiation in the visually-guided task, they may use remembering visual information in the memory-guided task. The recollection of memory might be reflected on the power signals before the reaching onset.

Contrary to our assumption that the higher-order cognitive processes affect the implicit adaptation, there was no difference in the after effect between the tasks. This result raises another possibility that the visually-guided task is related to both explicit and implicit information. Previous studies have suggested that activity in the frontal area is involved in explicitly modulating movements^13,16^. Consistent with this possibility, the Fz beta power was decreased in the visually-guided task before reaching initiation. Furthermore, the beta-band power in the parietal electrode was also decreased in the visually-guided task. Thus, the parietal cortex may contain direction-selective neurons, which could be altered by the visuomotor adaptation^46^. This means that the visually-guided task used in this study is driven by explicit modification. It is possible that subjects adjusted explicitly in the visually-guided task because the prediction error shown in the delta increase was reliable. Since the gradual rotation may not depend only on implicit adaptation, subjects needed to modulate explicitly for the error. Therefore, it is likely that the contribution of the explicit adaptation is greater in the visually-guided task while the implicit adaptation is at the same level between the tasks. We did not observe any difference in the after effect, but the retention rate in the adaptation block was higher in the visually-guided task. This discrepancy could be caused by the measuring method of the after effect. In the current study, we conducted the visually-guided trials without cursor rotation as the washout immediately after the adaptation trials, but this way might not be able to evaluate the after effect properly. A previous study implemented de-adaptation trials, which gave feedback in the opposite direction to the adaptation trials, after the adaptation trials. If subjects perform trials without perturbation after that, the recovery of adaptation behavior would be observed^47^. Hence, it is possible that implementation of de-adaptation trials reveals differences in the after effect between the tasks.

In summary, our findings suggest that the visually-guided task leads to higher adaptation and retention rates based on a reliable prediction error. Behavioral results indicated higher rates of adaptation and retention in the visually-guided task. Furthermore, the delta power associated with the prediction error increased for the visual feedback in the visually-guided task. Therefore, we suggest that the motor adaptation and the processing of the prediction error are affected by the task-type, such as the visually-guided and memory-guided tasks.

## Data Availability

The data that support the findings of this study are available from the corresponding author, upon reasonable request.
